# Molecular Evidence of *Plasmodium vivax* Mono and Mixed Malaria Parasite Infections in *Duffy*-Negative Native Cameroonians

**DOI:** 10.1371/journal.pone.0103262

**Published:** 2014-08-01

**Authors:** Huguette Gaelle Ngassa Mbenda, Aparup Das

**Affiliations:** Evolutionary Genomics and Bioinformatics Laboratory, Division of Genomics and Bioinformatics, National Institute of Malaria Research, Sector 8, Dwarka, New Delhi, India; Université Pierre et Marie Curie, France

## Abstract

The malaria parasite *Plasmodium vivax* is known to be majorly endemic to Asian and Latin American countries with no or very few reports of Africans infected with this parasite. Since the human *Duffy* antigens act as receptors for *P. vivax* to invade human RBCs and Africans are generally *Duffy*-negative, non-endemicity of *P. vivax* in Africa has been attributed to this fact. However, recent reports describing *P. vivax* infections in *Duffy*-negative Africans from West and Central parts of Africa have been surfaced including a recent report on *P. vivax* infection in native Cameroonians. In order to know if Cameroonians living in the southern regions are also susceptible to *P. vivax* infection, we collected finger-prick blood samples from 485 malarial symptomatic patients in five locations and followed PCR diagnostic assays with DNA sequencing of the 18S ribosomal RNA gene. Out of the 201 malaria positive cases detected, 193 were pure *P. falciparum*, six pure *P. vivax* and two mixed parasite infections (*P. falciparum* + *P. vivax*). The eight *P. vivax* infected samples (six single + two mixed) were further subjected to DNA sequencing of the *P. vivax* multidrug resistance 1 (*pvmdr1*) and the *P.vivax* circumsporozoite (*pvcsp*) genes. Alignment of the eight Cameroonian *pvmdr1* sequences with the reference sequence showed high sequence similarities, reconfirming *P. vivax* infection in all the eight patients. DNA sequencing of the *pvcsp* gene indicated all the eight *P. vivax* to be of VK247 type. Interestingly, DNA sequencing of a part of the human *Duffy* gene covering the promoter region in the eight *P. vivax*-infected Cameroonians to identify the T-33C mutation revealed all these patients as *Duffy*-negative. The results provide evidence of single *P. vivax* as well as mixed malaria parasite infection in native Cameroonians and add knowledge to the growing evidences of *P. vivax* infection in *Duffy*-negative Africans.

## Introduction

Malaria is a highly infectious vector-borne disease of the tropical and sub-tropical countries of the globe. Almost all the African countries are endemic to malaria, contributing about 90% of the total global malaria death incidences [1]. Apart from global effort to control malaria, this disease remains as a frontline infectious disease in Africa and in other malaria endemic regions of the globe. Relief and management of malaria majorly rely on treatment of infected patients by chemotherapy. Since as many as five different species of the genus *Plasmodium* (*Plasmodium falciparum*, *P. vivax*, *P. ovale*, *P. malariae* and *P. knowlesi*) are known to infect humans [2], [3] either singly or as mixed parasitic infections, proper diagnosis of specific malaria parasitic infection holds the key for effective treatment and management of malaria. Although microscopy is traditionally considered as gold standard for malaria diagnosis, in recent years, molecular diagnostic approaches by PCR (Polymerase Chain Reaction) assays have evolved as the most sensitive method to accurately diagnose single as well as mixed malaria parasite infections [4], [5], [6]. Several field epidemiological studies in different malaria endemic countries of the globe (including some African countries) [6], [7], have adapted this highly sensitive malaria diagnostic approach.

In recent years, malaria diagnosis by PCR assays have not only confirmed high incidences of malaria due to *P. falciparum* infections in Africa, but instances of *P. vivax* infections in *Duffy*-negative individuals have surfaced in many African countries, *e.g.*, Madagascar [9], Mauritania [12], Angola [11], Equatorial Guinea [11], Ethiopia [13]. Historically, *P. vivax* is known to be majorly prevalent in Asia and Latin America but largely absent in west and central African countries. This situation is explained by the fact that (i) the human *Duffy* Antigen Receptor for Chemokines (DARC) is used by *P. vivax* merozoites to invade the human RBCs [14], (ii) a mutation (T-33C) located in the promoter region of the human *Duffy* gene (otherwise known as *Duffy*-negative individuals) blocks the red blood cell invasion of *P. vivax*, and (iii) the T-33C mutation (causing *Duffy*-negativity) is nearly fixed in Africans [15], [16]. Since the *Duffy*-negative Africans are naturally protected from *P. vivax* infection, it has been proposed that the T-33C mutation has been selected in humans [17]. Considering the recent hypothesis on the African origin of *P. vivax* [18] it is quite possible that the fixation of the T-33C mutation might have been propelled by long exposure to *P. vivax* infection in Africans.

To this respect, Cameroon, a West-Central African country is inhabited mostly by *Duffy*-negative humans (95–99%) [19], with high prevalence of *P. falciparum* malaria (up to 100%) [20], [21]. Till 2013, incidences of *P. vivax* infection reported in Cameroon were restricted to non-native Cameroonians [22]. Very recently, it has been found that native Cameroonians (*Duffy* positive as well as *Duffy* negative individuals) were also able to be infected by the malaria parasite *P. vivax* [23]. Considering Cameroon as “Africa in miniature” with respect to malaria epidemiology [21], the recent report on native Cameroonians being infected with *P. vivax* [23] and the fact that Cameroon borders with malaria endemic countries (such as Equatorial Guinea) reporting *P. vivax* infection in *Duffy*-negative individuals, it is therefore important not only to survey the extent of malaria due to infection of different species of malaria parasites in native Cameroonians, but also to explore other regions of the country for the possible detection of *P. vivax* malaria infection. Since it has been hypothesized that the *Duffy*-positive Africans serve as reservoirs for *P. vivax* transmission [9], [11] and *Duffy*-positive native Cameroonians found to be infected by *P. vivax* [23], in case we find *P. vivax* infections in native Cameroonians, it will be of further interest to know the *Duffy*-status of the *P. vivax* infected patients.

## Material and Methods

### Ethics Statement

The present study has been approved by the Ethical Committee of Cameroon (*No003/CNE/SE/2012*) and written informed consents were obtained from all adult patients and the guardians of the minor patients.

### Sample collection and DNA isolation

**Figure 1:**
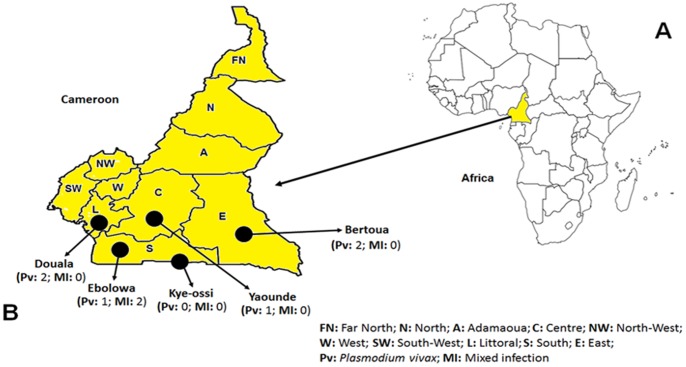
Map of Africa (A) highlighting Cameroon and (B) Map of Cameroon showing the sampling location sites.

We have collected finger-prick blood samples as 4–5 spots (each spot contains about 100 microliters of blood) on Whatman filter papers from 485 malaria symptomatic patients attending hospitals located in five different areas in southern Cameroon (Figure 1). The spots were dried and brought to the laboratory in New Delhi, India. In the laboratory, routine protocols were followed to isolate malaria parasite DNA from the blood spots using QIAamp mini DNA kit (Qiagen, Germany). Only one blood spot for each sample was used to isolate DNA at a time. The genomic DNA was eluted in a total volume of 80 µl of AE buffer (Qiagen, Germany).

### PCR-amplification of different genes

Since the conclusions drawn in the present study greatly relies on the molecular assays of different genetic fragments, PCR reactions were performed in four different genes (three genes of *Plasmodium* species and one of human). Details of the PCR conditions followed for specific genes are provided in the Tables S1 and S2. Whereas we used the published primers for amplification of the 18S rRNA gene of both *P. falciparum* and *P. vivax*, *pvmdr1*, *pfmdr1* and human *Duffy* genes, the primer sequences of the *pvcsp* gene were freshly designed in the present study. Although the general PCR conditions for all the genes were almost similar, different annealing temperatures were required for proper amplification of each of the genes (Tables S1 and S2).

For diagnosis of malaria parasites through PCR assay, we followed the nested PCR technique targeting the 18S rRNA gene of malaria parasites [4], [5]. This sensitive technique has been used in a number of studies [6], [11] and is suitable for differential identification of the four principal *Plasmodium* species associated with human malaria; *i.e*., *P. falciparum*, *P. vivax*, *P. malariae* and *P. ovale* as single or mixed infections. Following the published literature [4], [5], two sets of nested PCR reactions were performed for each sample; one for identification of *P. falciparum*, *P. vivax*, *P. malariae* and the other for *P. ovale* (Table S1). The DNA isolation (from a separate blood spot) and PCR amplification processes were repeated two more times for reconfirmation in samples presenting infections of *P. vivax* following similar protocol as described above.

In order to verify the PCR detection of *P. vivax* infection in Cameroonians based on the 18S rRNA gene, we have additionally PCR amplified two other genes of *P. vivax*; *pvmdr1* and *pvcsp*. Whereas the *pvmdr1* gene is associated with resistance to multiple antimalarial drugs (*pvmdr1*), the *pvcsp* is a highly polymorphic gene, commonly used in the characterization of *P. vivax* populations for different strain variants (VK210, VK247 and *P. vivax*-like) circulating around the world [24], [25]. The published primers [26] of *pvmdr1* gene amplify a 543 bp DNA fragment and the primers for *pvcsp* gene amplify a 507 bp product. The details of the PCR conditions of both the genes are presented in the Table S2. Similarly, in order to verify the presence of *P. falciparum* in the mixed infections (*P. vivax* + *P. falciparum*) as detected with PCR diagnostic assay with the 18S rRNA gene ([4], [5] see above), we PCR amplified a 510 bp DNA fragment of the *P. falciparum* multi-drug resistance gene 1 (*pfmdr1*) by nested PCR using the published primers [27], [28] (Table S2).

Since *P. vivax* is reported to infect *Duffy*-negative Africans [7], [11], [12], [13], with an interest to know if the *P. vivax*-infected Cameroonians are also *Duffy*-negative, we used the isolated DNA from samples presenting *P. vivax* infection (which also contain human genomic DNA) and used the published primers [29] to PCR amplify a fragment of ∼516 bp region covering the -33^rd^ nucleotide position (located in the promoter region) of the human *Duffy* gene [29]. We have followed this approach because, it is well known that DNA sequencing of the promoter region of the human *Duffy* gene with respect to the -33^rd^ nucleotide mutation can adeptly determine the *Duffy*-status of humans [11], [17], [29], [30]. This approach relies on the fact that finding the C nucleotide in a single peak in the DNA chromatogram at the -33^rd^ position specifies complete *Duffy*-negative status (also known as FY*O homozygote genotype), meaning no expression of the *Duffy* protein on the erythrocytes [31]. However, detection of both the nucleotides C and T (in double peak) at the same position depicts the heterozygote genotype and the T nucleotide in a single peak indicates the absence of FY*O genotype [29]. The details of the PCR protocols are provided in the Table S2. Alike the 18S rRNA gene, the PCR amplification processes for *pvmdr1*, *pfmdr1*, *pvcsp* and the human *Duffy* genes were repeated twice for the accurateness of identification/characterization of *P. vivax* species and determination of the *Duffy* status.

### Purification of PCR products, DNA sequencing and multiple DNA sequence alignments

In order to sequence and determine sequence identity/variations in the respective genes of the malaria parasites and Cameroonians with the respective reference sequences, we first purified the respective PCR products with Exonuclease-I and Shrimp Alkaline Phosphatase (Fermentas, Life Sciences) using the PCR thermal cycler (37°C for 1 hour and then 85°C for 15 minutes). The purified PCR products were then processed for DNA sequencing reactions with Big Dye Terminator as per standard protocol of the Applied Biosystems (ABI). The products were then run in the ABI 3730 XL DNA analyzer (in-house facilities of NIMR, New Delhi). For each DNA fragment, sequencing was performed from both the 3′ and 5′ directions (2X coverage). For each individual (human and parasites alike), separate contigs were formed with the two sequences from both the directions (3′ and 5′) using the SeqMan module of the DNASTAR (Madison, USA) computer program. Multiple sequences of each homologous gene of the malaria parasites (18S rRNA, *pvmdr1, pvcsp* and *pfmdr1*) and of the Cameroonians (*Duffy*) were aligned using the MegAlign module of the DNASTAR (Madison, USA) computer program to ascertain similarities and differences with the respective reference sequences. For example, the respective 18S rRNA gene sequences of *P. vivax* and *P. falciparum* were aligned with the reference sequences of *P. vivax* 18S rRNA gene of the *SAL*-1 strain (accession number U03079.1) and with the reference sequence of *P. falciparum* 18S rRNA gene of the 3D7 isolate (accession number AL844501), independently. Similarly, the *pvmdr1* and *pfmdr1* genes were aligned with the respective reference strains of *P. vivax* (*SAL*-1 strain, accession number XM_001613678) and *P. falciparum* (3D7 strain, accession number AL844504). Further, the human *Duffy* gene and *pvcsp* sequences generated in the present study were aligned with the respective reference sequences [accession numbers NG_011626.1 (*Duffy*) and GU339059.1 (*pvcsp SAL*-1 strain)]. All the references sequences (18S rRNA genes of *P. vivax* and *P. falciparum*, *pvmdr1* gene, *pfmdr1* gene, *pvcsp* and *Duffy* gene) were retrieved from the NCBI website (http://www.ncbi.nlm.nih.gov/) using the BLAST search.

## Results

**Figure 2:**
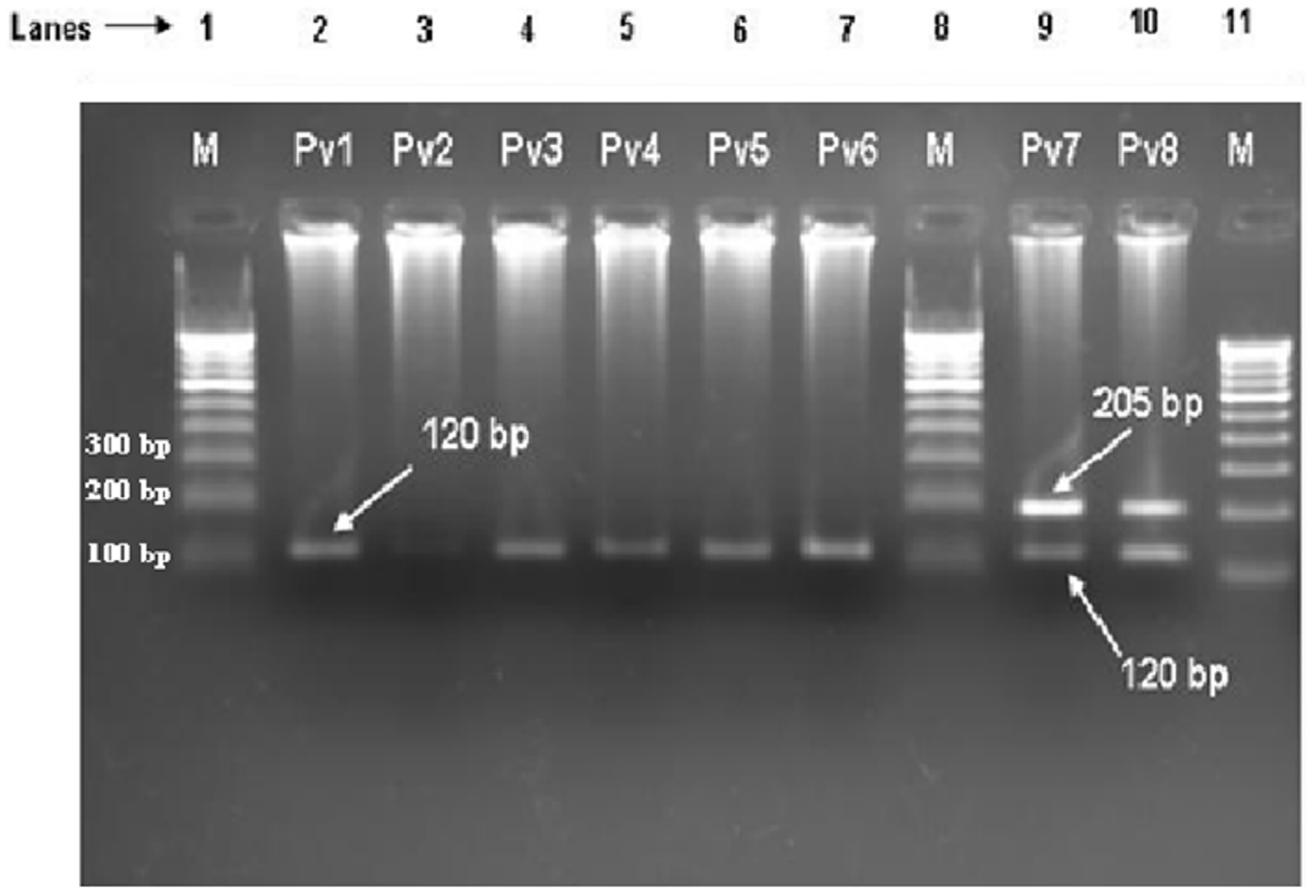
Gel picture showing bands of *P. vivax* mono (lanes 2–7) and mixed infection (lanes 9 and 10). Lanes 1, 8 and 11 were loaded with 100*P. vivax* and *P. falciparum*, respectively.

We have successfully isolated genomic DNA of malaria parasites for all the 485 blood samples from malaria symptomatic patients. Out of these 485 samples, only 201 were found to be infected with malaria parasites following PCR diagnostic assays of the 18S rRNA gene (Table 1). As expected, majorities of the infections (193, 96%) were due to *P. falciparum*. Interestingly, six patients (3%) were found to be infected by *P. vivax* only (Figure 2), and two cases (1%) of mixed parasitic infections (*P. falciparum* and *P. vivax*) were also detected (Figure 2). However, no single or mixed infections due to *P. malariae* or *P. ovale* could be identified in the present study. All the eight cases of *P. vivax* infections (six single and two mixed) were found in four different places in the southern Cameroon (Table 1). In order to confirm the results from the PCR analyses, different other genes of *P. vivax* and *P. falciparum* (*pvmdr1*, *pfmdr1* and *pvcsp*) were also PCR amplified and sequenced (see below).

**Table 1:** Patient details and the differential malaria species infection dynamics in Cameroon.

Sampling sites (sample size)	Gender (sample size)	Age Range (Minimum-Maximum)	Number of infected patients		
			*P. falciparum*	*P. vivax*	*P. falciparum + P. vivax*
Ebolowa (60)	Male (37)	(7 months–56 years)	36	0	1
	Female (23)	(1 month–82 years)	21	1	1
Douala (52)	Male (25)	(3 weeks–46 years)	25	0	0
	Female (27)	(9 months–80 years)	25	2	0
Bertoua (25)	Male (14)	(5 months–52 years)	13	1	0
	Female (11)	(1 year–50 years)	10	1	0
Yaounde (29)	Male (11)	(6 months–64 years)	10	1	0
	Female (18)	(1 years–78 years)	18	0	0
Kye-ossi (35)	Male (17)	(1 year–70 years)	17	0	0
	Female (18)	(1.7 year–65 years)	18	0	0

**Figure 3:**
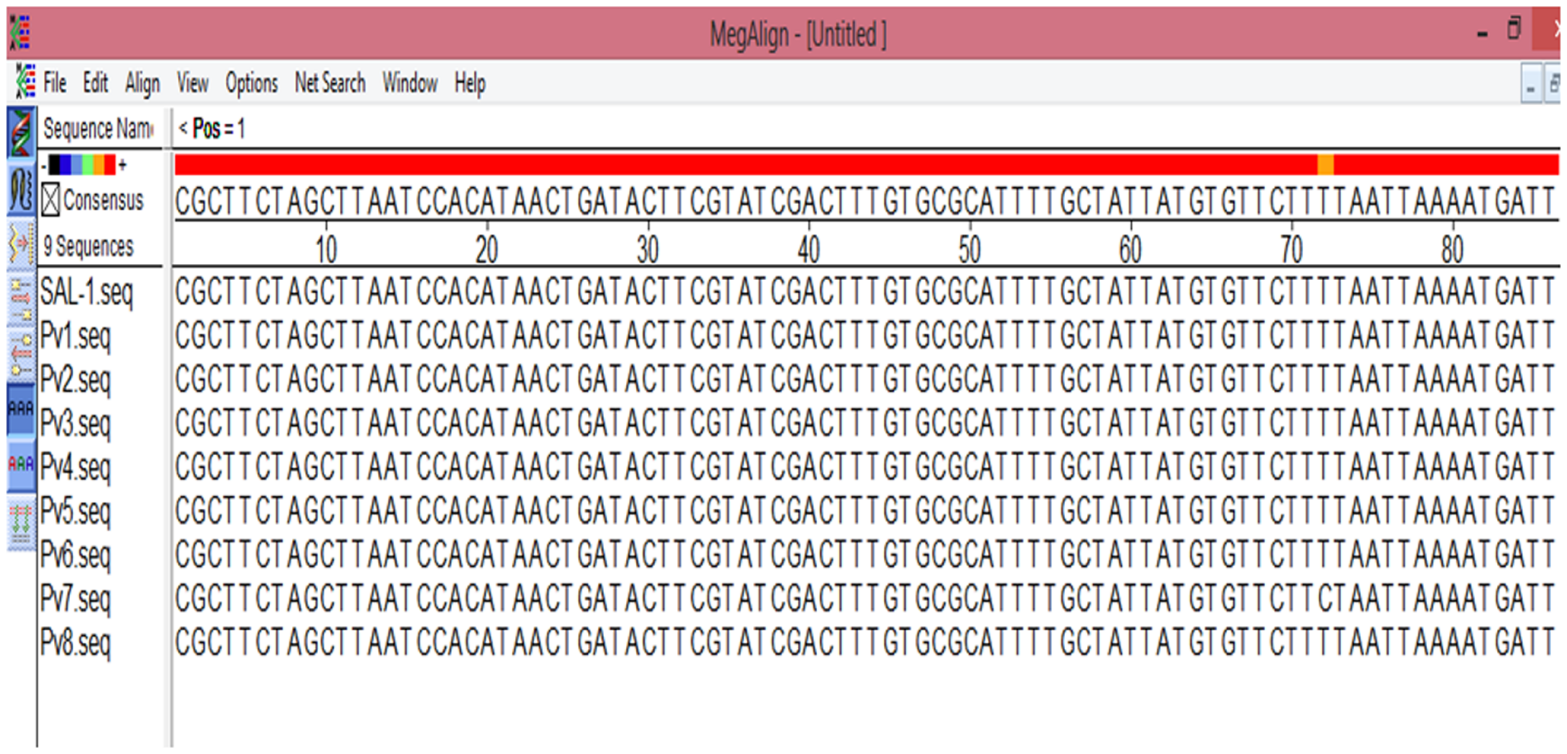
DNA sequence alignment of the eight Cameroonian *P. vivax* isolates with the reference DNA sequence of *P. vivax* SAL-1 strain.

**Figure 4:**
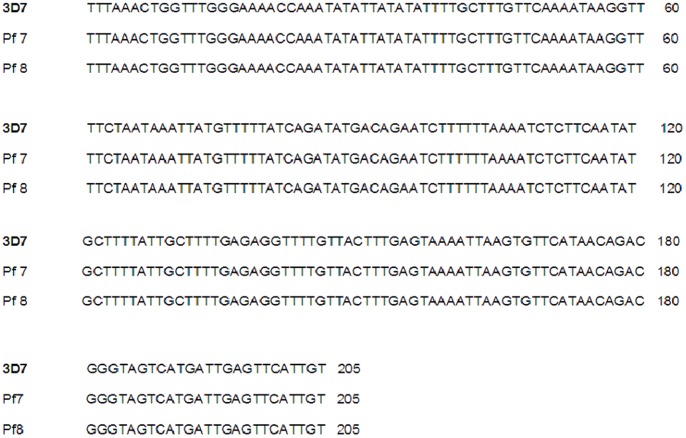
DNA sequence alignment of Cameroonian *P. falciparum* isolates from the mixed infection cases with the reference DNA sequence of *P. falciparum* 3D7 strain.

In order to substantiate the PCR assays results (as above) on the six incidences of single *P. vivax* and two cases of mixed infections (due to co-infection of *P. vivax* and *P. falciparum*), we have performed DNA sequencing of all the eight samples for different genes specific to *P. vivax* and *P. falciparum*. The sequencing of the eight 18S rRNA genes in *P. vivax* (six single and two mixed) followed by multiple sequences alignment of the eight 18S rRNA genes of *P. vivax* infected samples (six single and two mixed) and the reference sequence of the 18S rRNA gene (total nine sequences, Figure 3) and similar alignment of the two sequences of *P. falciparum* (mixed infection with *P. vivax*) and the *P. falciparum* reference sequence of the 18S rRNA (total three sequences, Figure 4) indicate perfect homology (98–100% similarities) between the DNA sequences generated in the present study with the respective reference sequences (Figures 3 and 4) for both *P. vivax* and *P. falciparum*. The newly generated sequences of the 18S rRNA genes of *P. vivax* have been deposited in the EMBL-Bank (accession numbers HF945436 to HF945443), and of *P. falciparum* in the GenBank (accession numbers KC428741 to KC428742).

With a view to corroborate the results on the detection of single *P. vivax* and the mixed parasitic infection as detected by PCR and DNA sequencing of the 18S rRNA genes, we have additionally sequenced the *pvmdr1* gene in the same eight samples (six single *P. vivax* infection and two mixed infection) and the *pfmdr1* gene in the two mixed infected samples. Apart from the presence of four nucleotide substitutions (three non-synonymous and one synonymous) (Figure S1), multiple sequence alignments for the *pvmdr1* genes with the respective reference sequences revealed perfect sequence homology between the reference *pvmdr1* gene of the *SAL*-1 strain and the eight *pvmdr1* sequences of Cameroonian *P. vivax*. For the *pfmdr1* gene too, apart from a single non-synonymous substitution (at the 86^th^ amino acid position) in one of the two Cameroonian *P. falciparum* isolates from the mixed parasitic infections with *P. vivax*, perfect sequence homology was observed when compared with the reference 3D7 *P. falciparum* strain. The results are presented in the Figures S1 and S2. The eight newly generated sequences of the *pvmdr1* and the two sequences of the *pfmdr1* gene have been deposited in the GenBank with accession numbers from KJ534638-KJ534645 and KJ534646-KJ534647, respectively.

With some evidences of *P. vivax* infection in Cameroonians using PCR diagnostic approaches followed by DNA sequencing and sequence alignments of the 18S rRNA and the *pvmdr1* genes, we were interested not only to reconfirm *P. vivax* infection, but also to know the type of *P. vivax* strain present in these eight Cameroonians. For this, we have sequenced a portion of the *pvcsp* gene covering the majority of the repeat regions in all the eight *P. vivax* isolates. Multiple sequence alignments with the reference sequence from the *SAL*-1 strain retrieved from the GenBank (accession number GU339059.1) showed that all the sequences from Cameroon display the non-apeptide repeats ANGA(G/D)(N/D)QPG, characteristic of the VK247 variant (Figure S3). These results therefore not only revalidated the observation of *P. vivax* infection in eight native Cameroonians, but also indicated that all the eight *P. vivax* are of VK247 type. The newly generated sequences of the *pvcsp* gene have been deposited in the GenBank (accession numbers KM099676 to KM099683).

**Figure 5:**
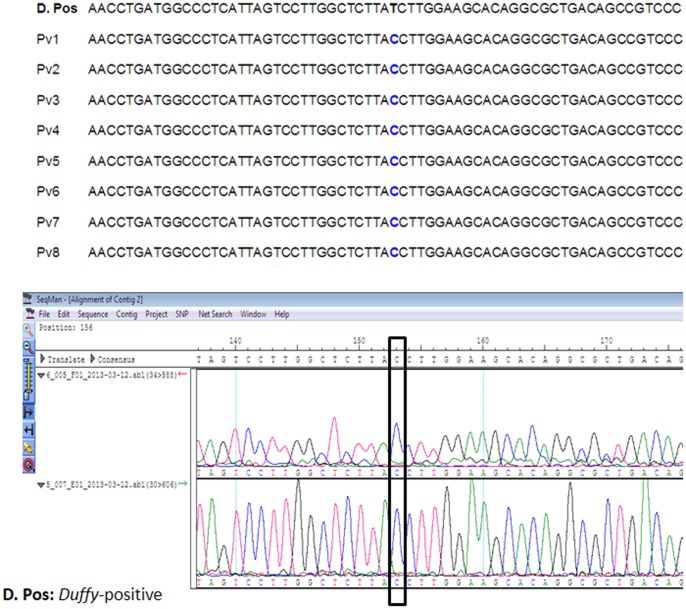
DNA sequence alignment and associated chromatogram of the DNA sequence covering the promoter region of the *Duffy* gene in eight Cameroonians harboring the *P. vivax* infection. The T-33C SNP is indicated in blue and the representative chromatogram showing a clear peak of “C” is shown below the alignment.

Since many African countries in the recent years report *P. vivax* malaria infection in *Duffy*-negative Africans, we tested this hypothesis by sequencing a region of the human *Duffy* gene covering the promoter region in the eight patients infected with *P. vivax*. Interestingly, we found the -33C mutation in all the eight patients, signifying the fact that all the eight native Cameroonians are *Duffy*-negative. In order to further discern if these eight Cameroonians are homozygous to the C mutation at the -33^rd^ position of the promoter region, we have carefully visualized and inspected the occurrence of single/double peaks at the T-33C nucleotide position in each of the two sequence chromatograms of each single Cameroonian (2X coverage, see above). Interestingly, for all the eight Cameroonians, we found single peak of C nucleotide at the -33^rd^ position of the promoter region of the human *Duffy* gene in both the sequences from the forward and reverse directions (Figure 5), signifying the fact that all the eight Cameroonians infected with either single *P. vivax* or mixed infection with *P. falciparum* are homozygous *Duffy*-negative. The eight newly generated sequences of the promoter region of the *Duffy* gene have been submitted at the GenBank (accession numbers from KJ534648-KJ534655).

## Discussion

Accurate diagnosis of malaria parasites holds the key for successful intervention through chemotherapy. To this respect, in recent years PCR-based malaria diagnosis has emerged as a gold standard technique for accurate identification of single as well as mixed malaria parasitic infections [4], [5], [6]. Identification of specific malaria parasite infection through PCR often is followed by DNA sequencing of the 18S rRNA gene for confirmatory purpose [6]. In many recent studies, some malaria parasite species-specific genes, *e.g. pvcsp*, *pvdhfr*, *etc*. have often been sequenced as additional markers for re-confirmatory purpose in different malaria endemic settings [10], [11].

**Figure 6:**
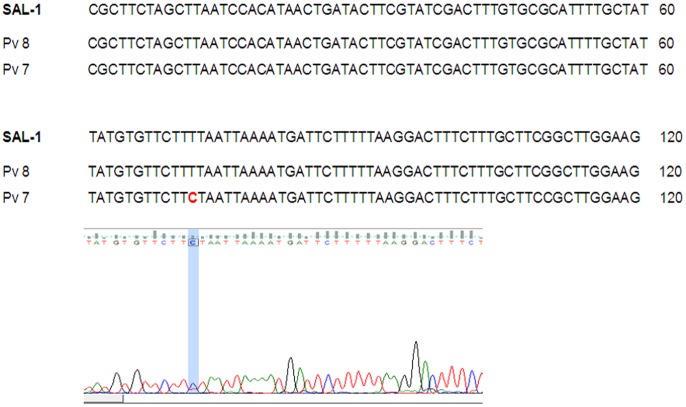
DNA sequence alignment of Cameroonian *P. vivax* isolates from the mixed infection cases with the reference DNA sequence of *P. vivax SAL*-1 isolate. The Single Nucleotide Polymorphism (SNP) in Pv7 is indicated in red and the representative chromatogram showing a single clear peak of “C” is shown below.

In the present study, PCR diagnostic assays with the 18S rRNA gene revealed the presence of 193 *P. falciparum*, six *P. vivax* and two mixed infections due to *P. falciparum* and *P. vivax* in Cameroon. The results of DNA sequencing and sequence alignments of the 18S rRNA gene therefore re-confirm the ability of the molecular diagnostic approach in detecting the single infections of *P. vivax* as well as mixed infection due to these two species by PCR amplification [4], [5], [6]. Although the DNA sequences of the 18S rRNA gene of Cameroonian *P. falciparum* yielded 100% similarity with the reference sequence, for *P. vivax*, when compared with the reference sequence of the *SAL*-1 strain, we found a novel single nucleotide polymorphism (SNP) at the 72^nd^ nucleotide position in one of the mixed-infected patients (Figure 6). Very similar results following analogous protocols (PCR diagnostic assays followed by DNA sequencing) could determine high incidences of mixed malaria parasite infection in India [6]. Taking together the results of the present study with the study from India [6], it could therefore be highlighted that nested PCR amplification of the malaria parasite 18S rRNA gene followed by DNA sequencing and multiple sequence alignment with the reference sequences of the respective 18S rRNA genes of malaria parasites could serve as a valuable aid for molecular diagnosis of malaria infection.

DNA sequencing and multiple sequence alignments of the *pvmdr1*, *pvcsp* and *pfmdr1* genes in the Cameroonian *P. vivax* and *P. falciparum* isolates could validate the results obtained with PCR diagnostic assay. While the four nucleotide substitutions in the *pvmdr1* gene in Cameroonian *P. vivax* might be population-specific in nature, the observed sequence homology of the *pvmdr1* gene in the present study with the reference *P. vivax SAL*-1 strain reconfirms the presence of *P. vivax* infection in native Cameroonians. The results on the whole on the DNA sequencing and sequence comparison with reference strains of the *pvmdr1* and *pfmdr1* genes therefore are in good agreement with the findings with the PCR diagnostic approach followed by DNA sequencing of the 18S rRNA gene (see above). It is thus recommended that associated DNA sequencing and sequence alignments of additional malaria parasite species-specific genetic markers should be conducted to confirm the observations with PCR diagnostic assay targeting the 18S region of the rRNA gene.

Genotyping of the *pvcsp* gene encompassing the repeats region is often considered to differentiate among three different strains circulating in the global *P. vivax* populations [32]. To this context, the *P. vivax* VK210 strain type (also called classic type) is known to be distributed in higher frequency in comparison to the VK247 strain in almost all *P. vivax* endemic regions [32]. However, in some countries (including African countries) where genotyping of the *pvcsp* gene has been performed, such as India, Afghanistan, Angola, Equatorial Guinea, both the *pvcsp* variants (VK210 and VK247) have been found to be circulating in different localities of the same country [11], [33], [34]. Finding of *P. vivax* strains of only VK247 type in Cameroon in the present study is therefore interesting. Considering the limited number of *P. vivax* samples in the present study, such a conclusion (on distribution of only VK247 strain in Cameroon) should be dealt with caution; genotyping more number of isolates will ascertain the distribution of specific strain types in Cameroonian *P. vivax*.

The *Duffy*-status of eight Cameroonians infected with *P. vivax* could be ascertained by direct sequencing after PCR amplification of the part of the human *Duffy* gene covering the promoter region with the -33^rd^ nucleotide position. Very similar approach has been followed in studies in Indians [29] and Ethiopians [13] in successfully determining the *Duffy* status of humans. Finding of all the eight Cameroonians homozygous for the mutated C nucleotide (-33C) justifies that all the eight Cameroonians infected with *P. vivax* are homozygous *Duffy*-negative, and that *P. vivax* can infect *Duffy*-negative Cameroonians [23] and to other Africans [7], [9], [11], [12], [13]. The present observation on the *P. vivax* infection in *Duffy*-negative Cameroonians together with similar findings in other African countries therefore indicate that *P. vivax* might have evolved other, hitherto unknown mechanism(s), to infect *Duffy*-negative humans. Considering the emerging hypothesis on the African origin of *P. vivax* [18], it seems highly likely that Africans in general have been selected for the *Duffy*-negative mutation due to long exposure to *P. vivax* infection. However, since the current rate of transmission of *P. vivax* is fairly low in Africa in general, and Cameroon in particular, other host-genetic factor(s) might also be playing significant role in *P. vivax* infection in *Duffy*-negative host genetic background. Furthermore, two out of the five principal malaria vectors of Africa (*Anopheles gambiae* and *An. arabiensis*) responsible for majority of malaria transmission in Cameroon have been reported to have high vectorial capacity to transmit *P. vivax* malaria parasite [7], [35]. Since *P. vivax* infection in asymptomatic native Cameroonians has been reported in the south-western part of the country [23], and the fact that the southern part of Cameroon borders Equatorial Guinea and incidences of *P. vivax* infection in indigenous human populations have already been reported in Equatorial Guinea [11], it seems imperative that *P. vivax* parasites are capable of successful transmission in multiple African countries. Although the exact mechanism leading to successful infection of *P. vivax* to *Duffy*-negative Africans has not yet been established, it seems for now that *P. vivax* infection might have been mediated independently (or in combination) of factors like, (i) evolution and spread of specific *P. vivax* strain capable of infecting the *Duffy*-negative humans, (ii) host genetic susceptibility and (iii) vectorial competence. Considering the simple genomic architecture, high mutation rate, less generation time (in comparison to humans and mosquitoes), it seems highly likely that the *P. vivax* strains circulating in Sub-Saharan Africa might have evolved to the extent for being able to infect the *Duffy*-negative Africans. However, whether such *P. vivax* strains are specific to Africa or introduced from other *P. vivax* malaria endemic countries is not known. More in-depth sampling of malaria parasites from Sub-Saharan Africa and following population genomic studies with multiple putatively neutral SNPs already developed in this malaria parasite [36], [37] could unravel the evolutionary history of African *P. vivax*.

How important is the report on *P. vivax* infection in *Duffy*-negative humans for the malaria control programme of Cameroon? Cameroon, like other African countries is already struggling to control malaria due to *P. falciparum*; additional burden of *P. vivax* mono-infection as well as *P. vivax*-*P. falciparum* mixed infection is therefore daunting. Furthermore, *P. vivax* infections were found to be spread in four out of five places of collection in the southern parts of Cameroon, which are already reeling under high malaria mortality coupled with chloroquine resistant *P. falciparum* [21]. This is exemplified by the recent finding on the occurrence of multiple *pfcrt* haplotypes conferring chloroquine-resistance in Cameroonian *P. falciparum* [38]. The situation is especially griming considering the infection capabilities of *P. vivax* in *Duffy*-negative Cameroonians and other Africans [7], [9], [11], [12], [13]. As recommended by the World Health Organization, the national treatment policy in Cameroon has established artemisinin-based combination therapies (ACTs; artesunate + amodiaquine [AS+AQ] and artemeter + lumefantrine [AL]) in 2006 as the first-line treatment for uncomplicated malaria [39], [40]. Clearly, the current malaria drug policy in Cameroon does not take consideration of either single *P. vivax* infection or mixed parasite infections [1].

## Supporting Information

Figure S1 **Multiple sequences alignment for** ***pvmdr1*** **gene.**
(TIF)

Figure S2 **Multiple sequences alignment for** ***pfmdr1*** **in the two isolates mixed infected (** ***P. vivax*** **+** ***P. falciparum*** **).**
(TIF)

Figure S3 **Alignment of Cameroonian** ***pvcsp*** **sequences with the reference sequence of SAL-1 strain.**
(TIF)

Table S1 **PCR protocol and cycling conditions used for the malaria diagnostic for** ***P. falciparum*** **,** ***P. vivax*** **,** ***P. malariae*** **and** ***P. ovale*** **.**
(DOCX)

Table S2 **PCR cycling conditions used for the** ***pvmdr1*** **,** ***pfmdr1*** **,** ***pvcsp*** **and** ***Duffy*** **genes.**
(DOCX)

## References

[1] World Health Organization (2013) World malaria report: 2013.

[2] Singh B, Kim SL, Matusop A, Radhakrishnan A, Shamsul SS, et al (2004) A large focus of naturally acquired *Plasmodium knowlesi* infections in human beings. Lancet 363: 1017–1024. 15051281 10.1016/S0140-6736(04)15836-4

[3] Tyagi RK, Das MK, Singh SS, Sharma YD (2013) Discordance in drug resistance-associated mutation patterns in marker genes of *Plasmodium falciparum* and *Plasmodium knowlesi* during coinfections. J Antimicrob Chemother 68: 1081–1088. 23292346 10.1093/jac/dks508

[4] Snounou G, Viriyakosol S, Zhu XP, Jarra W, Pinheiro L, et al (1993) High sensitivity of detection of human malaria parasites by the use of nested polymerase chain reaction. Mol Biochem Parasitol 61: 315–320. 8264734 10.1016/0166-6851(93)90077-b

[5] Johnston SP, Pieniazek NJ, Xayavong MV, Slemenda SB, Wilkins PP, et al (2006) PCR as a confirmatory technique for laboratory diagnosis of malaria. J Clin Microbiol 44: 1087–1089. 16517900 10.1128/JCM.44.3.1087-1089.2006 PMC1393165

[6] Gupta B, Gupta P, Sharma A, Singh V, Dash AP, et al (2010) High proportion of mixed-species *Plasmodium* infections in India revealed by PCR diagnostic assay. Trop Med Inter Health 15: 819–824. 10.1111/j.1365-3156.2010.02549.x 20487427

[7] Ryan JR, Stoute JA, Amon J, Dunton RF, Mtalib R, et al (2006) Evidence for transmission of *Plasmodium vivax* among a *Duffy* antigen negative population in western Kenya. Am J Trop Med Hyg 75: 575–581. 17038676

[8] Culleton R, Ndounga M, Zeyrek FY, Coban C, Casimiro PN, et al (2009) Evidence for the transmission of *Plasmodium vivax* in the Republic of the Congo, West Central Africa. J Infect Dis 200: 1565–1569. 10.1086/644510 19803728

[9] Ménard D, Barnadas C, Bouchier C, Henry-Halldin C, Gray LR, et al (2010) *Plasmodium vivax* clinical malaria is commonly observed in *Duffy*-negative Malagasy people. Proc Natl Acad Sci USA 107: 5967–5971. 20231434 10.1073/pnas.0912496107 PMC2851935

[10] Dhorda M, Nyehangane D, Rénia L, Piola P, Guerin PJ, et al (2011) Transmission of *Plasmodium vivax* in south-western Uganda: report of three cases in pregnant women. PLoS One 6: 19801. 10.1371/journal.pone.0019801 PMC3094453 21603649

[11] Mendes C, Dias F, Figueiredo J, Mora VG, Cano J, et al (2011) *Duffy* negative antigen is no longer a barrier to *Plasmodium vivax*-molecular evidences from the African West Coast (Angola and Equatorial Guinea). Plos Negl Trop Dis 5: e1192. 21713024 10.1371/journal.pntd.0001192 PMC3119644

[12] Wurtz N, Mint LK, Bogreau H, Pradines B, Rogier C, et al (2011) *Vivax* malaria in Mauritania includes infection of a *Duffy* negative individual. Malar J 10: 336. 22050867 10.1186/1475-2875-10-336 PMC3228859

[13] Woldearegai TG, Kremsner PG, Kun JF, Mordmuller B (2013) *Plasmodium vivax* malaria in *Duffy*-negative individuals from Ethiopia. Trans R Soc Trop Med Hyg 107: 328–331. 23584375 10.1093/trstmh/trt016

[14] Miller LH, Mason SJ, Clyde DF, McGinniss MH (1976) The resistance factor to *Plasmodium vivax* in blacks. The *Duffy*-blood-group genotype, FyFy. New Engl J Med 295: 302–304. 778616 10.1056/NEJM197608052950602

[15] Mendis K, Sina BJ, Marchesini P, Carter R (2001) The neglected burden of *Plasmodium vivax* malaria. Am J Trop Med Hyg 64: 97–106. 11425182 10.4269/ajtmh.2001.64.97

[16] Gething PW, Elyazar IRF, Moyes CL, Smith DL, Battle KE, et al (2012) A long neglected world malaria map: *Plasmodium vivax* endemicity in 2010. PLoS Negl Trop Dis 6: e1814. 22970336 10.1371/journal.pntd.0001814 PMC3435256

[17] Hamblin MT, Di-Rienzo A (2000) Detection of the signature of natural selection in humans: evidence from the *Duffy* blood group locus. Am J Hum Genet 66: 1669–1679. 10762551 10.1086/302879 PMC1378024

[18] Liu W, Li Y, Shaw KS, Learn GH, Plenderleith LJ, et al (2014) African origin of the malaria parasite *Plasmodium vivax* . Nat Commun 5: 3346 DOI: 10.1038 24557500 10.1038/ncomms4346 PMC4089193

[19] Culleton RL, Mita T, Ndounga M, Unger H, Cravo PV, et al (2008) Failure to detect *Plasmodium vivax* in west and central africa by PCR species typing. Malar J 7: 174. 18783630 10.1186/1475-2875-7-174 PMC2546428

[20] World Health Organization (2012) World malaria report: 2012.

[21] Ngassa MHG, Awasthi G, Singh PK, Gouado I, Das A (2014) Does malaria epidemiology project Cameroon as “Africa in Miniature”? J Bios 39: 1–11. 10.1007/s12038-014-9451-y 25116627

[22] Guerra CA, Howes RE, Patil AP, Gething PW, Van Boeckel TP, et al (2010) The international limits and population at risk of *Plasmodium vivax* transmission in 2009. Plos Negl Trop Dis 4: e774. 20689816 10.1371/journal.pntd.0000774 PMC2914753

[23] Fru-Cho J, Bumah VV, Safeukui I, Nkuo-Akenji T, Titanji PKV, et al (2014) Molecular typing reveals substantial *Plasmodium vivax* infection in asymptomatic adults in a rural area of Cameroon. Malar J 13: 170. 24886496 10.1186/1475-2875-13-170 PMC4032583

[24] Imwong M, Pukrittayakamee S, Grüner AC, Rénia L, Letourneur F, et al (2005) Practical PCR genotyping protocols for *Plasmodium vivax* using Pvcs and Pvmsp-1. Malar J 4: 20. 15854233 10.1186/1475-2875-4-20 PMC1131918

[25] Qari SH, Shi YP, Goldman IF, Udhayakumar V, Alpers MP, et al (1993a) Identification of *Plasmodium vivax*-like human malaria parasite. Lancet 341: 780–783. 8095999 10.1016/0140-6736(93)90559-y

[26] Lekweiry KM, Ali Ould Mohamed Salem B, Gaillard T, Wurtz N, Bogreau H, et al (2012) Molecular surveillance of drug-resistant *Plasmodium vivax* using pvdhfr, pvdhps and pvmdr1 markers in Nouakchott, Mauritania. J Antimicrob Chemother 67: 367–374. 22086859 10.1093/jac/dkr464

[27] Vathsala PG, Pramanik A, Dhanasekaran S, Devi CU, Pillai CR, et al (2004) Widespread occurrence of the *Plasmodium falciparum* chloroquine resistance transporter (*Pfcrt*) gene haplotype SVMNT in *P. falciparum malaria* in India. Am. J. Trop. Med. Hyg. 70: 256–259. 15031513

[28] Basco LK, Ringwald P (2002) Molecular epidemiology of malaria in Cameroon. X. Evaluation of PFMDR1 mutations as genetic markers for resistance to amino alcohols and artemisinin derivatives. Am J Trop Med Hyg 66: 667–671. 12224572 10.4269/ajtmh.2002.66.667

[29] Chittoria A, Mohanty S, Jaiswal YK, Das A (2012) Natural selection mediated association of the *Duffy* (*FY*) gene polymorphisms with *Plasmodium vivax* malaria in India. PLoS ONE 7: e45219. 23028857 10.1371/journal.pone.0045219 PMC3448599

[30] Hamblin MT, Thompson EE, Di Rienzo A (2002) Complex signatures of natural selection at the Duffy blood group locus. Am J Hum Genet 70: 369–383. 11753822 10.1086/338628 PMC419988

[31] Tournamille C, Colin Y, Cartron JP, Le Van Kim C (1995) Disruption of a GATA motif in the Duffy gene promoter abolishes erythroid gene expression in Duffy-negative individuals. Nat Genet 10: 224–228. 7663520 10.1038/ng0695-224

[32] de Souza-Neiras WC, de Melo LM, Machado RL (2007) The genetic diversity of *Plasmodium vivax*: a review. Mem Inst Oswaldo Cruz 102: 245–254. 17568928 10.1590/s0074-02762007000300002

[33] Kim JR, Imwong M, Nandy A, Chotivanich K, Nontprasert A, et al (2006) Genetic diversity of *Plasmodium vivax* in Kolkata, India. Malaria J 5: 71. 10.1186/1475-2875-5-71 PMC1560144 16907979

[34] Dhangadamajhi G, Rout BK, Kar SK, Ranjit MR (2010) Genetic diversity of *Plasmodium vivax* in a hyperendemic area predominated by *Plasmodium falciparum*; a preliminary study. Trop Biomed 27: 578–584. 21399600

[35] Taye A, Hadis M, Adugna N, Tilahun D, Wirtz RA (2006) Biting behavior and *Plasmodium* infection rates of *Anopheles arabiensis* from Sille, Ethiopia. Acta Trop 97: 50–54. 16171769 10.1016/j.actatropica.2005.08.002

[36] Gupta B, Dash AP, Shrivastava N, Das A (2010) Single nucleotide polymorphisms, putatively neutral DNA markers and population genetic parameters in Indian *Plasmodium vivax* isolates. Parasitol 137: 1721–1730. 10.1017/S0031182010000533 20594376

[37] Gupta B, Srivastava N, Das A (2012) Inferring the evolutionary history of Indian *Plasmodium vivax* from population genetic analyses of multilocus nuclear DNA fragments. Mol Ecol 21: 1597–1616. 22353169 10.1111/j.1365-294X.2012.05480.x

[38] Ngassa MHG, Das A (2014) Occurrence of multiple chloroquine-resistant *Pfcrt* haplotypes and emergence of the S(agt)VMNT type in Cameroonian *Plasmodium falciparum* . J Antimicrob Chemother 69: 400–403. 24092656 10.1093/jac/dkt388

[39] World Health Organization (2006) Guidelines for the treatment of malaria. Geneva, Switzerland: WHO.

[40] National Malaria Control Programme (2007) National guidelines for the treatment of malaria. Yaounde, Cameroon: National Malaria Control Programme.

