# Supplementary figures and images for: Molecular Evidence of Plasmodium vivax Mono and Mixed Malaria Parasite Infections in Duffy-Negative Native Cameroonians
Source: PLoS One. 2014 Aug 1;9(8):e103262. doi: 10.1371/journal.pone.0103262 (PMC4118857; doi:10.1371/journal.pone.0103262)

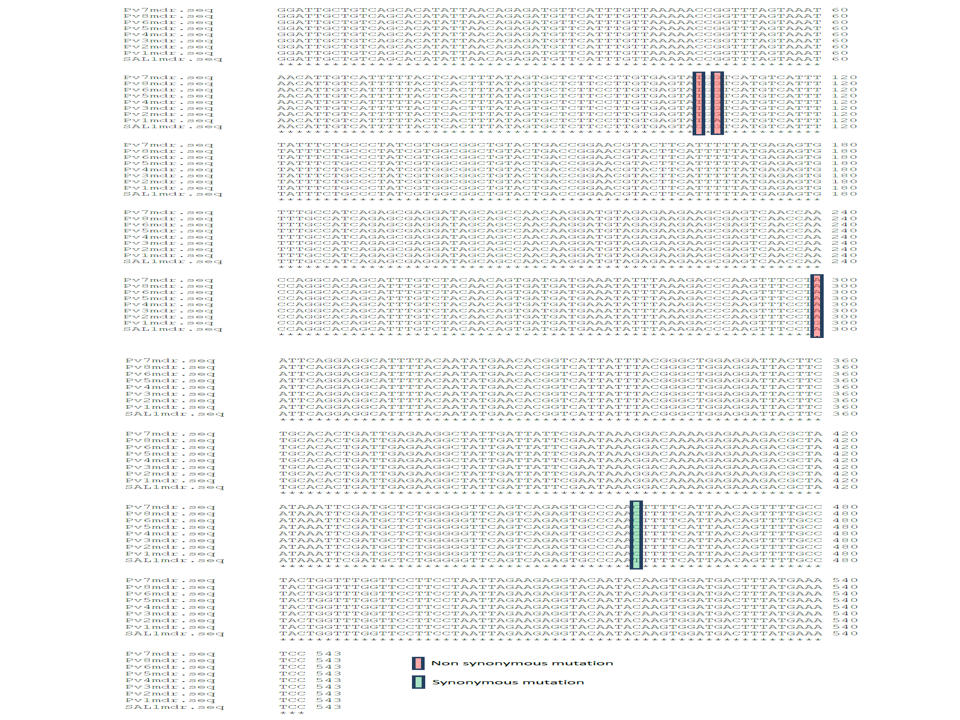

Supplement: Figure S1 — Multiple sequences alignment for pvmdr1 gene. (TIF) [file pone.0103262.s001.tif]

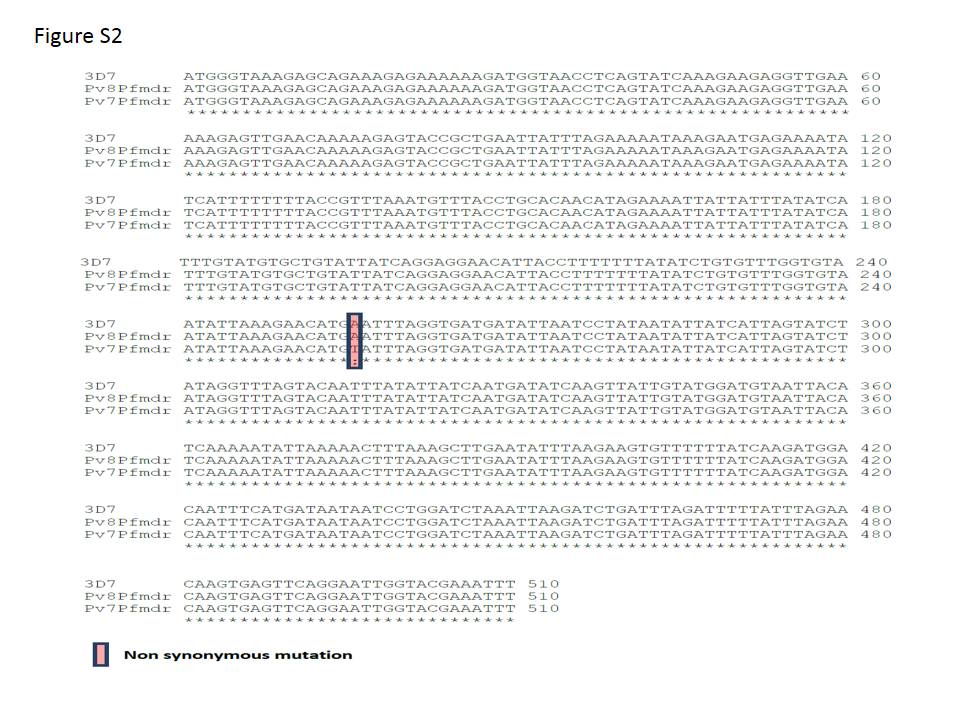

Supplement: Figure S2 — Multiple sequences alignment for pfmdr1 in the two isolates mixed infected ( P. vivax + P. falciparum ). (TIF) [file pone.0103262.s002.tif]

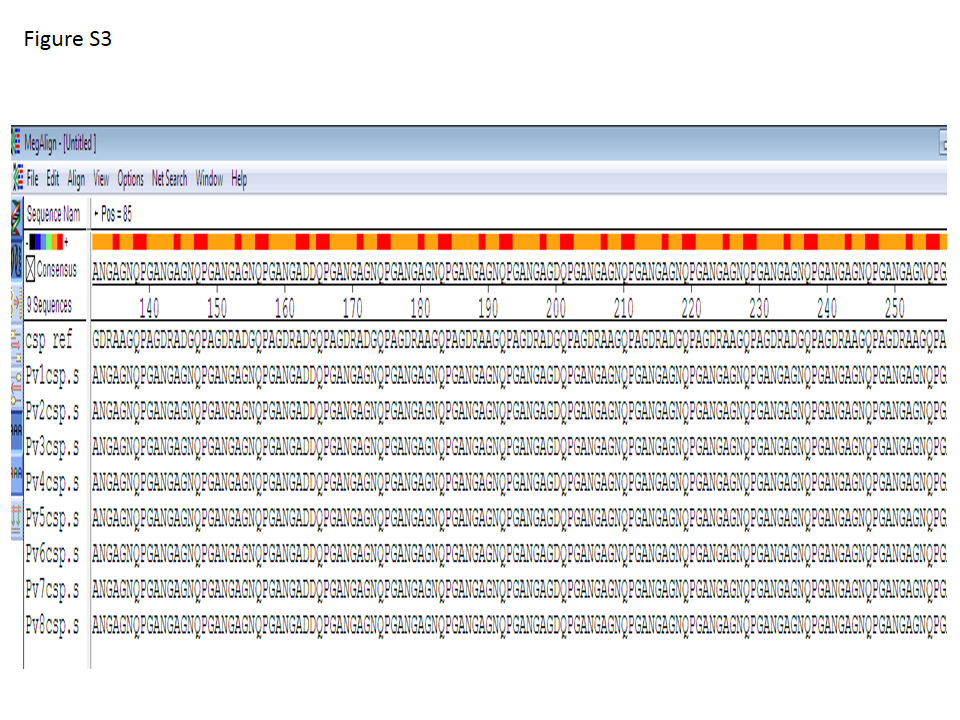

Supplement: Figure S3 — Alignment of Cameroonian pvcsp sequences with the reference sequence of SAL-1 strain. (TIF) [file pone.0103262.s003.tif]
