# Supplementary material for: Molecular Evidence of Plasmodium vivax Mono and Mixed Malaria Parasite Infections in Duffy-Negative Native Cameroonians
Source: PLoS One. 2014 Aug 1;9(8):e103262. doi: 10.1371/journal.pone.0103262 (PMC4118857; doi:10.1371/journal.pone.0103262)
Supplement: Table S1 — PCR protocol and cycling conditions used for the malaria diagnostic for P. falciparum , P. vivax , P. malariae and P. ovale . (DOCX) [file pone.0103262.s004.docx]

Table S1. PCR protocol and cycling conditions used for the malaria diagnostic for *P. falciparum*, *P. vivax,* *P. malariae* and *P. ovale*

|  | **Ingredients** | ***Pf*, *Pv*, *Pm*** | **Cycling conditions** | **Ingredients** | ***Po*** | **Cycling conditions** |
| --- | --- | --- | --- | --- | --- | --- |
| **1^st^ PCR** | Buffer | 2.5 µl | Initial denaturation: 94°C for 5 | Buffer | 2.5 µl | Initial denaturation: 94°C for 5 |
|  | dNTPs | 0.5 µl | minutes | dNTPs | 0.5 µl | minutes |
|  | 25µM primer 1^st^ step Forward | 0.4 µl | Start cycle: 30 cycles | 25µM primer 1^st^ step Forward | 0.4 µl | Start cycle: 30 cycles |
|  | 25µM primer 1^st^ step Reverse | 0.4 µl | Denaturation: 94°C for 1 minute | 25µM primer 1^st^ step Reverse | 0.4 µl | Denaturation: 94°C for 30 seconds |
|  | DNA template | 1 µl | Annealing: 60°C for 2 minutes | DNA template | 1 µl | Annealing: 45°C for 30 seconds |
|  | Taq Polymerase | 0.33 µl | Extension: 72°C for 2 minutes | Taq Polymerase | 0.33 µl | Extension: 72°C for 1 minute 30 |
|  | Water | 19.87 µl | End cycle | Water | 19.87 µl | seconds |
|  | Total volume | 25 µl | Final extension: 72°C for 10 minutes | Total volume | 25 µl | End cycle |
|  |  |  |  |  |  | Final extension: 72°C for 10 minutes |
| **2^nd^ PCR** | Buffer | 2.5 µl | Initial denaturation: 94°C for 5 | Buffer | 2.5 µl | Initial denaturation: 94°C for 5 |
|  | dNTPs | 0.5 µl | minutes | dNTPs | 0.5 µl | minutes |
|  | PCR product of the 1^st^ step | 3 µl | Start cycle: 30 cycles | PCR product of the 1^st^ step | 3 µl | Start cycle: 45 cycles |
|  | 25µM primer (Pf) Forward | 0.4 µl | Denaturation: 94°C for 1 minute | 25µM primer (Po) Forward | 0.4 µl | Denaturation: 94°C for 30 seconds |
|  | 25µM primer (Pf) Reverse | 0.4 µl | Annealing: 55°C for 2 minutes | 25µM primer (Po) Reverse | 0.4 µl | Annealing: 45°C for 30 seconds |
|  | 25µM primer (Pv) Forward | 0.4 µl | Extension: 72°C for 2 minutes | - | - | Extension: 72°C for 1 minute 30 |
|  | 25µM primer (Pv) Reverse | 0.4 µl | End cycle | - | - | seconds |
|  | 25µM primer (Pm) Forward | 0.4 µl | Final extension: 72°C for 10 minutes | - | - | End cycle |
|  | 25µM primer (Pm) Reverse | 0.4 µl |  | - | - | Final extension: 72°C for 10 |
|  | Taq Polymerase | 0.33 µl |  | Taq Polymerase | 0.33 µl | minutes |
|  | Water | 16.27 µl |  | Water | 17.87 µl |  |
|  | Total volume | 25 µl |  | Total volume | 25 µl |  |
