# Supplementary material for: Molecular Evidence of Plasmodium vivax Mono and Mixed Malaria Parasite Infections in Duffy-Negative Native Cameroonians
Source: PLoS One. 2014 Aug 1;9(8):e103262. doi: 10.1371/journal.pone.0103262 (PMC4118857; doi:10.1371/journal.pone.0103262)
Supplement: Table S2 — PCR cycling conditions used for the pvmdr1 , pfmdr1 , pvcsp and Duffy genes. (DOCX) [file pone.0103262.s005.docx]

Table S2. PCR cycling conditions used for the *pvmdr1*, *pfmdr1*, *pvcsp* and *Duffy* genes

|  | **Reactions** | **Cycling conditions** | **Amplicons size (bp)** |
| --- | --- | --- | --- |
| ***pvmdr1*** | 1^st^ PCR | Initial denaturation: 94°C for 5 minutes; Start cycle: 45 cycles (Denaturation: 94°C for 50 seconds; Annealing: 53°C for 1 minute; Extension: 72°C for 1 minute) End cycle; Final extension: 72°C for 10 minutes | 543 |
|  | 2^nd^ PCR | Initial denaturation: 94°C for 5 minutes; Start cycle: 45 cycles (Denaturation: 94°C for 50 seconds; Annealing: gradient temperature from 55°C to 65°C for 1 minute; Extension: 72°C for 1 minute) End cycle; Final extension: 72°C for 10 minutes | 543 |
| ***pfmdr1*** | 1^st^ PCR | Initial denaturation: 95°C for 5 minutes; Start cycle: 35 cycles (Denaturation: 95°C for 50 seconds; Annealing: 58.8°C for 1minute; Extension: 72°C for 1 minute) End cycle; Final extension: 72°C for 5 minutes | 510 |
|  | 2^nd^ PCR | Initial denaturation: 95°C for 5 minutes; Start cycle: 35 cycles (Denaturation: 95°C for 50 seconds; Annealing: 58.8°C for 1minute; Extension: 72°C for 1 minute) End cycle; Final extension: 72°C for 5 minutes | 510 |
| ***pvcsp*** |  | Initial denaturation: 95°C for 5 minutes; Start cycle: 35 cycles (Denaturation: 95°C for 1 minute; Annealing: 61.3°C for 1minute; Extension: 72°C for 1 minute) End cycle; Final extension: 72°C for 10 minutes | 507 |
| ***Duffy*** |  | Initial denaturation: 95°C for 5 minutes; Start cycle: 35 cycles (Denaturation: 95°C for 30 seconds; Annealing: 64°C for 30 seconds; Extension: 72°C for 30 seconds) End cycle; Final extension: 72°C for 5 minutes | 516 |
